# From Pine Cones to Read Clouds: Rescaffolding the Megagenome of Sugar Pine (*Pinus lambertiana*)

**DOI:** 10.1534/g3.117.040055

**Published:** 2017-04-05

**Authors:** Marc W. Crepeau, Charles H. Langley, Kristian A. Stevens

**Affiliations:** Department of Evolution and Ecology, University of California, Davis, California 95616

**Keywords:** 10× genomics, conifer genomes, genome assembly, sugar pine

## Abstract

We investigate the utility and scalability of new read cloud technologies to improve the draft genome assemblies of the colossal, and largely repetitive, genomes of conifers. Synthetic long read technologies have existed in various forms as a means of reducing complexity and resolving repeats since the outset of genome assembly. Recently, technologies that combine subhaploid pools of high molecular weight DNA with barcoding on a massive scale have brought new efficiencies to sample preparation and data generation. When combined with inexpensive light shotgun sequencing, the resulting data can be used to scaffold large genomes. The protocol is efficient enough to consider routinely for even the largest genomes. Conifers represent the largest reference genome projects executed to date. The largest of these is that of the conifer *Pinus lambertiana* (sugar pine), with a genome size of 31 billion bp. In this paper, we report on the molecular and computational protocols for scaffolding the *P. lambertiana* genome using the library technology from 10× Genomics. At 247,000 bp, the NG50 of the existing reference sequence is the highest scaffold contiguity among the currently published conifer assemblies; this new assembly’s NG50 is 1.94 million bp, an eightfold increase.

As a class, conifer genome reference sequences represent the largest genome assemblies accomplished to date. Recent and active conifer genome projects have target genomes far exceeding that of the human genome (Birol *et al.* 2013; Nystedt *et al.* 2013; Neale *et al.* 2014; Warren *et al.* 2015; Stevens *et al.* 2016). Most of the size of conifer genomes derives from insertions of transposable elements (Nystedt *et al.* 2013; Stevens *et al.* 2016). Their large scale and repetitive nature make them an important assembly benchmark, and a target for technological improvements. The largest genome project to date is that of the white pine *Pinus lambertiana* (sugar pine), with a genome size of 31 Gbp (Stevens *et al.* 2016).

Complexity reduction played an important role in the initial assembly of *P. lambertiana*. A gymnosperm megagametophyte (MGP) is maternally derived tissue found within each seed containing the same haploid genome that is contributed to the diploid zygote (embryo). The haploid MGP contains enough DNA for creation of a set of size-selected paired end Illumina libraries. These can be sequenced to sufficient depth to support the creation of a high quality, error corrected, read set forming the basis for *de novo* assemblies (Zimin *et al.* 2014; Stevens *et al.* 2016). In this paper, we present a protocol for scaffolding a conifer genome applying the new library technology, described in detail below, from 10× genomics (www.10xgenomics.com). Because DNA requirements are small, the protocol can be implemented using DNA from a single conifer megagametophyte. Application of this new protocol to *P. lambertiana* results in an eightfold improvement to the scaffold contiguity of the colossal genome. For most MGPs, extracted DNA will be of sufficient quality and quantity that the protocol presented here can be used in parallel with methods for generating standard size selected paired end libraries (Zimin *et al.* 2014).

The idea of reducing the complexity of the assembly problem by simplifying the target has been around since the early genome projects. The shotgun sequencing and assembly of cloned DNA was the original method of constructing “synthetic long reads”. On a larger scale, the shotgun sequencing and assembly of fosmid pools to create “synthetic long reads” has also been used effectively for the assembly of complex genomes (Duitama *et al.* 2012; Zhang *et al.* 2012) and proposed for conifers (Alexeyenko *et al.* 2014). Alternatively, Illumina’s TruSeq Synthetic Long Read technology (formerly Moleculo) uses shotgun sequencing and assembly of amplified high molecular weight (HMW) DNA to construct synthetic long reads (Voskoboynik *et al.* 2013). These protocols suffer a common practical limitation on the number of independent subhaploid pools that can be generated. Contiguity information is extracted from each pool by deep sequencing and assembly to generate synthetic long reads, which are useful as inputs to another assembly.

Recently, it has become practical to replace the deep sequence coverage requirement of synthetic long reads, with a deep subhaploid pool coverage. We follow Kuleshov *et al.* (2016) and refer to the resulting data as synthetic long read “clouds.” When aligned to a genome, each long DNA fragment is represented by a low-density cloud of clustered reads. Currently, few methods exist for implementing deep pool coverage.

The GemCode system from 10× Genomics (www.10xgenomics.com) utilizes a combination of proprietary hardware and reagents to produce Illumina sequencing libraries that preserve the long-range linkage information present in large DNA molecules. The two key components of their platform are (1) a microfluidic device designed to produce emulsified droplets each containing a small “pool” of HMW DNA fragments, and (2) distinct indices drawn from a massive set (∼750,000) of 14-bp oligonucleotide “barcodes” (see Figure 1). Each library begins with ∼1 ng of input DNA, which is partitioned into ∼200,000 barcoded pools. These droplet partitions are proprietarily named GEMs, for Gel bead in EMulsion. Because the amount of partitioned DNA is small (<10 fg), for genomes of sufficient size, each GEM will contain a collection of genomic fragments comprising a small fraction of the haploid genome. Multiple libraries can be used to further increase the total number of barcoded pools generated. High quality linkage information is contingent upon HMW input DNA. One advantage of the 10× Genomics protocol, in this regard, is a relatively small amount of input DNA (∼1 ng) recommended.

**Figure 1:**
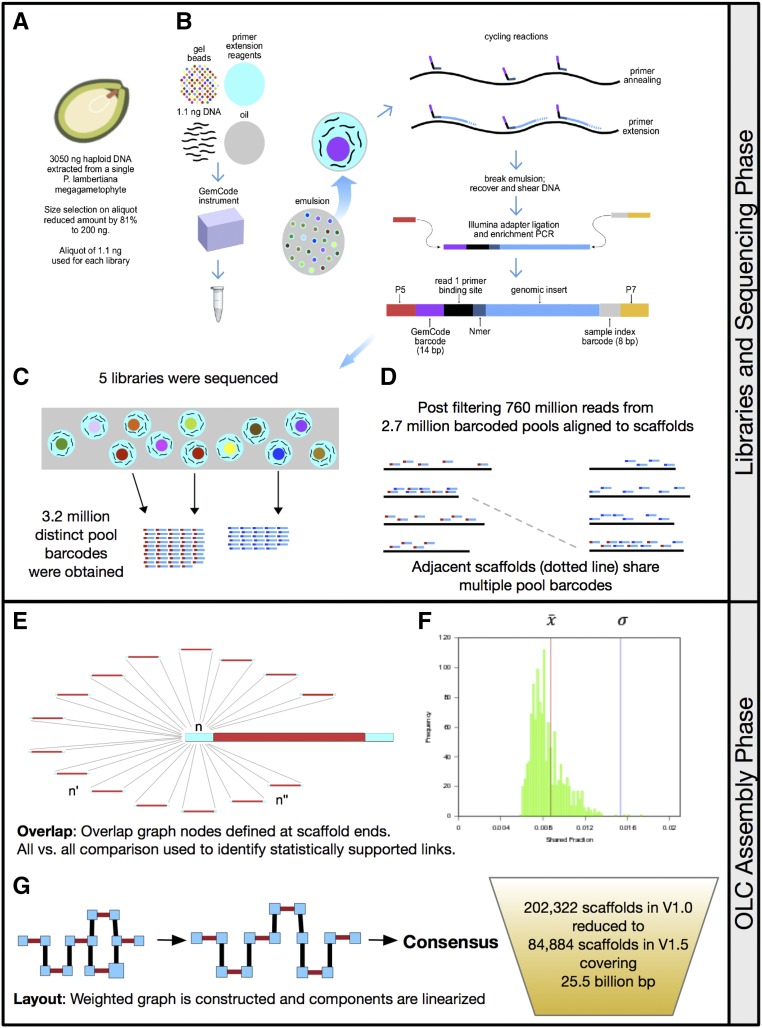
(Top) Construction of synthetic long read clouds with 10× Genomics technology. (A) HMW DNA is prepared from a single haploid sugar pine MGP. (B) Within the instrument emulsion droplets are used to pool HMW DNA. A barcode containing bead in each droplet is used for indexing the pools. During subsequent thermal cycling the bead dissolves and the input DNA fragments act as templates for primer extension by the barcoded random hexamers (blue). All extension products within a droplet contain the same 14 bp barcode (magenta). After completion of primer extension cycles the emulsion is “broken,” pooled extension products are physically sheared to subkilobase fragments, and p7 and p5 adapters are added to the barcoded terminal fragments by ligation and enrichment PCR. Final molecules have the standard configuration and adapter sequences of Illumina dual-index, paired-end libraries, except that i5 is 14 bp instead of 8 bp. The random hexamer sequence (Nmer) is trimmed from the start of read one during data processing. (C) The oligo-containing gel bead (colored dots) within each droplet contains a single 14 bp barcode and multiple long DNA fragments (wavy lines) that serve as templates for generation of library fragments and then reads (short bicolored bars). Different droplets may contain identical gel beads increasing the effective size of the pool. All reads with the same barcode form an effective pool. (D) Alignments to a contig will assign the contig to one or more pools. Overlap graph nodes are defined in windows at the contig ends. Because there are a large number of distinct pools (barcodes), any two nodes are unlikely to belong to the same two (or more) pools by chance. However, one shared barcode is common. (Bottom) Scaffolding with synthetic long read clouds and fragScaff. (E) For each node, the read group assignments for that node are compared with the read group assignments for every other node and the fraction of read groups shared between them (their “shared fraction”) is calculated. (F) A typical distribution of shared fraction for a node in our data. For each node, a normal distribution is fitted to the observed shared fraction data. Link scores for each ordered pair of nodes are computed by taking the negative log_10_ probability of the observed shared fraction under the fitted normal distribution. After all pairwise link scores are calculated, a global link score threshold is determined. In our example, the pair (n, n′) shared one barcode and are below the threshold, while the pair (n, n′′) shared >1 and are above. (F) Pairs of nodes with a score exceeding the global link score threshold become edges in an overlap graph with a weight corresponding to the score. (G) Layout linearizes the weighted graph and is accomplished by greedy algorithm described in (Adey *et al.* 2014). From each linearized subgraph the consensus scaffold is determined by concatenating component scaffolds.

The first application for this data was haplotype phasing, which relies on a reference genome alignment to directly demarcate the long (linking) DNA fragments from aligned reads. This follows similar implementations by others using related (but lower-plexity) data types (Peters *et al.* 2012; Amini *et al.* 2014). Utilizing read clouds for assembly relies on a property of the interactions between >1 barcode. For the subhaploid dilution achieved, multiple DNA fragments have the same barcode, hence any given barcode is associated with many parts of the genome. However, the dilution into pools is of sufficient magnitude to ensure that coincidental overlap between two barcodes is rare. This implies that two (or more) barcodes will intersect at a unique location. Similarly, if two or more barcodes are shared between unlinked sequences, they are likely in close proximity to each other. Few programs exist that will utilize this information for genome assembly (Adey *et al.* 2014; Kuleshov *et al.* 2016). To create our new assembly we used the *fragScaff* program from Adey *et al.* (2014). *fragScaff* implements a modification of the classic overlap–layout–consensus paradigm (Myers 2005). Specifically, *fragScaff* uses a link score to define overlaps and build an overlap graph. Each edge in this graph has a weight. The layout step is accomplished by finding a new simpler graph, which, when traversed, defines a scaffold. The consensus step concatenates component scaffold sequences with spacers.

The primary input to *fragScaff* is a bam formatted alignment file. In the case of our data, each aligned read is annotated with a 14 bp barcode. Sequencing may be single read, for our scaffolding protocol, the additional read in paired-end sequencing is used only to help resolve alignment location. The other important input is a bed formatted annotation of repetitive regions of the genome, which is used to establish the subset of trusted alignments. We address methods for creating this annotation as well as setting the most important assembly parameters in this paper.

Our results build on the results of Mostovoy *et al.* (2016) for the human genome. We demonstrate the utility and scalability of this approach, and establish a protocol for the much larger, more repetitive, and more fragmented genomes of conifers. We validate our approach using empirical estimates of the type 1 and type 2 error rates in linking.

## Materials and Methods

### Library construction and sequencing

DNA was extracted from a single sugar pine MGP using the method previously reported in Zimin *et al.* (2014) for obtaining HMW DNA from pine needles. In total, 3.1 µg of DNA was obtained. Size selection was performed using an aliquot of 1.1 µg run on a PAC30KB BluePippin cassette (Sage Science, Inc.) with a 40 kb cutoff, yielding 200 ng of size-selected DNA. Library SPX1 was constructed to evaluate non-size-selected DNA while libraries SPX2 through SPX5 used the size-selected DNA. All libraries began with 1.2 ng of DNA, and followed the protocol in the version B GemCode user guide with the shearing regime adapted to the Covaris E220 (Covaris, Inc.). GemCode DNA was brought to a final volume of 130 µl and sheared in a Covaris eight microTUBE strip at the Covaris E220 recommended settings for 800 bp fragments. DNA was then concentrated to 50 µl using 1.8× SPRI-select beads (Beckman-Coulter, Inc.).

For evaluation purposes, we initially sequenced one non-size-selected library (SPX1), and one size-selected library (SPX2) on two lanes of a HiSeq2500 Rapid Run flowcell with a dual-index, pair-end run regime of 98+8+14+98 cycles. The size-selected library showed a substantial improvement in the overall distribution of fragment sizes and physical coverage (Table 1). Subsequent effort focused on SPX2, and three additional size-selected libraries (SPX3, SPX4, and SPX5). These were sequenced on two lanes of a HiSeq4000 flowcell with a dual-index, pair-end run regime of 101+8+14+101 cycles.

**Table 1:** Library and sequencing results

(A) Sequence Statistics by Library					
Library	Paired Reads	Read Length (bp)	Raw Sequence Coverage (*X*)	Aligned Reads	Filtered Aligned Reads
1	232,879,210	88 + 98	0.7	181,462,035	111,854,014
2	232,329,746	88 + 98	0.7	195,340,154	125,001,615
3	311,708,094	91 + 101	0.97	288,643,022	177,242,573
4	297,865,798	91 + 101	0.92	274,488,516	168,110,243
5	310,580,224	91 + 101	0.96	286,623,246	177,826,361

(B) Barcoded Pool Statistics by Library					
Library	Barcodes (Pools)	Filtered Barcodes (Pools)	Physical Coverage (Gb)	Mean Read Cloud Length (kb)	*N*50 Read Cloud Length (kb)
1	609,077	546,857	73 (2.3×)	35.3	65
2	576,842	514,523	113 (3.6×)	61.2	80
3	663,880	556,645	204 (6.6×)	58.9	80
4	665,787	556,329	172 (5.6×)	57.6	75
5	665,787	562,421	177 (5.7×)	61.7	75

(A) Sequencing results are presented for each of the five libraries. A total of 4.25× sequence coverage of the 31 Gb genome was obtained. After barcode demultiplexing reads were aligned to *P. lambertiana* v1.0 with BWA and subsequently filtered by fragScaff (see *Materials and Methods*). (B) Pool statistics are presented for each library; >3.18 million barcoded pools were sequenced across all libraries. The mean read cloud length for HMW DNA covered ∼60 kb for all size-selected libraries, nearly twice as long as without size selection. We estimated the total physical coverage in read clouds to be 23.8×.

### Sequence processing and genome alignment

The Illumina BCL files for all sequencing runs were translated to FASTQ using *bclprocessor* (10× Genomics). The resulting FASTQ was demultiplexed (both sample indices and pool barcodes) using the BASIC pipeline (10× Genomics). Demultiplexed reads are labeled by a pool barcode: 14 bp barcode concatenated to a sample designator. A readgroups.tsv file was also produced containing all pool barcodes with 50 or more reads. Only barcodes contained this file were considered for scaffolding. Demultiplexed FASTQ files were aligned to the genome to be scaffolded using BWA (Li *et al.* 2010) specifying that read group identifiers, when present, be appended to each record (bwa mem -h readgroups.tsv). This output was then filtered with *samtools* to remove low quality alignments, PCR duplicates, unmapped reads, unpaired reads, and supplemental alignments (samtools view -q 10 -f 2 –F 3084).

### Read cloud length and physical coverage estimation

We used the read cloud length distribution as a proxy for the molecular weight distribution of the fragments used for library construction. We demarcated read clouds for purposes of estimating a length distribution. Reads aligning to the larger *P. lambertiana* v1.0 scaffolds (>500 kb) were isolated and the alignments were subsequently binned into 5 kb windows across each scaffold. Consecutive runs of windows with the same barcode were interpreted as read clouds. We expect, on average, the read clouds containing multiple windows will be padded by one half window on either end. To compensate for this we subtracted one window width from all our estimates. To estimate density, the total number of reads contained in the read clouds defined for each library was divided by the sum of the read cloud lengths in kilobase for that library to determine the aligned reads per kilobase. To estimate genome wide physical coverage, the observed physical coverage from the subset of larger scaffolds was scaled to the complete genome using the ratio of all aligned reads to reads aligned to the larger scaffolds. This scaling factor was consistently estimated as 3.1.

### Repeat annotation

*fragScaff* employs BED formatted files to ignore repeats and ambiguous bases in the definition of nodes. A RepeatMasker exclusion file was prepared using the *P. lambertiana* v1.0 repeat annotation described in Stevens *et al.* (2016). A mapping-quality-based exclusion file was prepared by converting low quality alignments (MAPQ ≤ 10) into BED formatted genomic intervals. These intervals were subsequently unioned with *bedtools* (Quinlan and Hall 2010) to form a nonredundant mask. Intervals were unioned if they were separated by <40 bp. The BED file annotating the coordinates of all intervals of Ns in the reference genome was created using a Python script.

### Calibration runs

To construct a dataset appropriate for calibration and validation, we split *P. lambertiana* v1.0 scaffolds at least 500 kb in length into windows that were 50 kb in length (excluding Ns) with the exception of the final window. This process generated 157,873 new *child* scaffolds from 10,403 *parent* scaffolds. These new *child* scaffolds were used as a validation dataset, replacing their parent scaffolds as the input data for *fragScaff*. Using a Python script, the bam and bed files were also transformed to the new coordinate system. Reads were reassigned to scaffolds based on their leftmost coordinate. Calibration run results were analyzed to compare the order of the reassembled child scaffolds with their ordering in the original *P. lambertiana* v1.0 parent scaffolds. The joins made by fragScaff and the potential joins were classified into the following mutually exclusive categories:

**Correct:** A join between two adjacent child scaffolds originating from the same parent scaffold.
**Type 1 error:** A join between two nonadjacent child scaffolds. If from different parents, only one child can be the first or last (*i.e.*, terminal) piece of its parent scaffold. Joins between nonterminal child scaffolds and smaller un-split scaffolds were also counted.
**Type 2 error**: Two unjoined child scaffolds that are adjacent in a parent scaffold.
**Ignored:** A join of two child scaffolds where both are terminal pieces from different parent scaffolds. These terminal joins between parents were considered unverifiable.

### Production run

Following calibration, we assembled original unmodified scaffolds using selected parameters. The *fragScaff* program was run with an updated terminal window size (–*E* = 10,000), clipping the top 1.5% of shared fraction distribution (–*D* = 0.985), and our custom repeat mask based on analysis of mapping quality. Furthermore, to improve tractability we limited *fragScaff* to the scaffolds 5 kb and longer (–*m* = 5000) representing 74.8% of the genome. The sparse coverage meant that many of the shortest genome elements would not have enough read group assignments to supply the statistical power necessary for inclusion in the assembly.

### Fosmid pool comparison

The program nucmer (nucmer–maxmatch –l 100 –c 100; delta-filter –r –i 90; Kurtz *et al.* 2004) was also used to align our assembly (*P. lambertiana* v1.0) to the collection of PacBio sequenced and assembled fosmids reported in Stevens *et al.* (2016). We expected a few fosmids to overlap joins made here in constructing *P. lambertiana* v1.5. Alignments involving joined scaffolds were inspected for evidence supporting or contradicting the joins.

### Data availability

The sequence data used for this study was deposited in the NCBI trace archive under BioProject 174450 and accession number SRX2629912. The assembly is available at the Pine Reference Sequence project site http://dendrome.ucdavis.edu/ftp/Genome_Data/genome/pinerefseq/Pila/v1.5/.

## Results

### Libraries and sequencing

We prepared five GemCode libraries from DNA extracted from a single *P. lambertiana* megagametophyte (Figure 1 and Table 1). The megagametophyte used was from the same mother tree (tree 5038; Stevens *et al.* 2016) as the *P. lambertiana* v1.0 reference genome assembly. The haplotype captured in our libraries is a sibling meiotic haplotype to the one used for *P. lambertiana* v1.0. We expect that 50% of the content will be the identical genotype.

In total, we sequenced 1,385,363,072 reads from barcoded libraries, representing 4.25× raw genome coverage. Subsequent alignment to the genome yielded 1,226,556,973 aligned reads containing valid barcodes. After Samtools filtering (see *Materials and Methods*), the total number of aligned reads with valid barcodes was further reduced to 760,034,806 (Table 1). For each library, more than half a million valid pool barcodes were present among the filtered reads; however, we only utilized alignments from barcodes present 50 or more times, which reduced the number of “read groups” (*i.e.*, distinct barcodes) per library to less than a quarter million (Table 1). The distribution of filtered alignments per pool was consistent across libraries with a negligible tail.

Across all size selected libraries, our estimated median length of read clouds was consistently ∼50 kb (Table 1) for the size-selected libraries. The *N*50 weighted averages for HMW DNA fragment length were also consistent, with half of the coverage coming from fragments > 80 kb (Table 1). The average number of alignments per kilobase within each read cloud varied from a low of 0.87 reads per kilobase for SPX3 to a high of 1.5 reads per kilobase for SPX1, with a median of 1.0 for SPX5. While read coverage was sparse (4.5×), total physical coverage in HMW DNA fragments was deeper (23.8×) for the haploid 31 Gbp genome.

### Parameter calibration and optimization

We deemed it critical to empirically validate our assembly results. We opted to use the most contiguous elements of our existing genome assembly as the internal control, and to generate a corresponding test dataset *in silico*. Specifically, we began with all 10,403 elements whose total length (including gaps) was ≥500 kb, and broke these into 157,873 pieces that were exactly 50 kb in length (excluding Ns) with the exception of the 3′ terminal pieces. These fragmented scaffolds then became the input data for *fragScaff*. The original order and orientation of the 50 kb pieces allowed us to evaluate the performance of our scaffolding procedure under different parameter settings. We refer to these assemblies as “calibration runs” since we used them to evaluate parameters for our final assembly of unmodified data.

This methodology relies on two plausible assumptions: (1) the existing assembly for elements >500 kb in length is essentially correct and representative; (2) the performance of the methodology for joining 50 kb elements will be indicative of the performance for the overall genome. It is also noteworthy that, because the haploid genome of our megagametophyte is not identical to the reference genome sequence, there is the possibility that genuine structural variation is a source of error.

Runs were performed in four stages: (1) bam file parsing, which included definition of nodes and barcode hits to each node; (2) shared fraction calculations, which was the most computationally intensive step; and (3) graph based layout and (4) consensus. We focused our efforts on evaluating and optimizing the exposed *fragScaff* parameters and procedures that directly effect the definition of nodes and edges in initial graph construction.

#### Optimizing repeat exclusion:

The *P. lambertiana* genome is highly repetitive. Reads originating from repetitive regions obfuscate scaffolding because their genomic locations are ambiguous. Hence, the fragScaff protocol recommends the specification of an exclusion file in BED format. Regions specified in this file will be excluded from nodes so that reads aligning within them are not considered. For the *P. lambertiana* genome, a repeat mask file masks 79% of the genome (Stevens *et al.* 2016). We ran a parameter-calibration run using this exclusion file with default parameters and estimated a type I error of 5.84%, and an even higher type II error of 25.73%, *i.e.*, only 74.27% of expected joins were made.

An alternative approach was to rely on BWA mapping quality estimates to annotate repetitive regions. For each alignment, BWA estimates a confidence in the location, in the form of a Phred transformed probability that the locus is correct (mapping quality or MAPQ). By default, the program only considers alignments with a mapping quality >10. We created an additional exclusion file based on the mapping quality estimates. This was done by collecting all alignments with MAPQ ≤ 10, converting the alignment coordinates to BED format (see *Materials and Methods*). This produced a less restrictive (more targeted) exclusion file. Similarly, using this exclusion file in a parameter-calibration run, we found that it improved type I error to 4.92%, while the type II error rate and contiguity were substantially improved: 84.80% of expected joins were made.

#### Optimizing the window size for defining graph nodes:

We evaluated two settings for the number of unmasked bases defining an overlap graph node (–*E* parameter), the default value of 5000 bp, and larger value of 10,000 bp, which would incorporate more barcodes. In both cases the maximum node length (–*o*) was kept at 50,000 bp, and the minimum input element length (–*m*) was set to 5000 bp. The median number of barcodes per node increased from 26 to 39. This increase improved the number of valid links made. Upon updating the value of –*E* from 5000 to 10,000, our estimated type II error rate dropped from 15.20 to 13.52%. This modest change has a substantial impact on the *N*50 value of the calibration run, increasing it by 25%. Our type I error rate increased slightly from 4.92 to 5.19%.

#### Optimizing overlap graph construction:

For each node, fragScaff generates a histogram of shared valid pool barcodes with all other nodes. This distribution forms the basis of the link score calculation, and ultimately what edges get included in the overlap graph. By default, the outlying 5% tails of this distribution is completely ignored. We noticed that many more false negatives than false positives fell in the right tail of this distribution. By examining the results of our parameter calibration runs, we were able to modify the value of the (–*D*) parameter leading to improved type II error rates relative to its default setting. Using calibration data, we estimated the likely impact of adjustments to the type I error and type II error rates. This led to updating of the parameter to filter the top 1.5% of the distribution, rejecting nodes with 98 or more read groups. Comparing similar calibration runs before and after parameter update, we observe a reduction in type II error rate from 13.52 to 8.69%, while type I error rate stayed relatively contained (5.19% *vs.* 4.85%, respectively).

### Final assembly and validation

The production fragScaff assembly of all *P. lambertiana* v1.0 elements ≥5 kb resulted in an eightfold improvement in scaffold *N*50 relative to the input (Table 2). Merging this product with the remaining (<5 kb) elements of *P. lambertiana* v1.0, we obtain a final *P. lambertiana* v1.5 reference genome. Scaling by a genome size of 31 billion bp, we obtain a scaffold NG50 of 1.94 million bp, this is also an eightfold improvement over the original NG50 scaffold of 247 kb.

**Table 2:** Assembly statistics before and after rescaffolding with long read clouds using fragScaff

	Original Assembly (with *N*’s)	Original Assembly (No *N*’s)	Rescaffolded Assembly (with *N*’s)	Rescaffolded Assembly (no *N*’s)
Maximum scaffold length (bp)	4,064,336	3,809,096	23,976,851	22,367,058
*N*50	324,201	306,897	2,668,366	2,509,905
*N*10	959,930	904,501	8,710,993	8,182,563
*N*90	72,460	69,664	406,554	399,889

The weighted average assembly length (*N*50) increased by ∼ eightfold. This was regardless of whether or not padding (*N*’s) was included in the calculation.

The final estimates of the type I and type II linking error rates correspond to the values of 4.85 and 8.69% reported for our final calibration run. We also validated links using an independent PacBio sequenced and HGAP assembled pool of 40 kb fosmid clones from the same diploid mother. We found 14 instances where nucmer results (see *Materials and Methods*) supported the overlap of two whole genome shotgun (WGS) elements by a single fosmid (although the highly repetitive content of the genome causes frequent spurious alignments that add uncertainty to such interpretations). Since the linking range of our GemCode data easily spans the length of a fosmid clone, we expected pairs of WGS elements contained within a fosmid to be ideal candidates for joining, and, indeed, 7 of the 14 were found to be joined in the *P. lambertiana* v1.5 assembly.

## Discussion

*P. lambertiana* is the largest genome sequenced and assembled to date. Among conifers, this reference genome assembly already stands out as having the highest scaffold contiguity (Nystedt *et al.* 2013; Neale *et al.* 2014; Warren *et al.* 2015; Stevens *et al.* 2016). Like most initial assemblies of megagenomes, large-scale scaffolding is the obvious next step toward a more contiguous and serviceable resource. In this paper, we report the generation of deep physical coverage from subhaploid pools using a single molecule sequencing technology (10× Genomics). Relatively light sequencing of the resulting libraries was needed to obtain a substantial eightfold increase to the weighted scaffold contiguity.

System performance with this technology is highly dependent on the quality, especially the size distribution, of the input DNA. In particular, the range of the linkage information produced is contingent upon the distribution of molecular weight of the starting DNA. We demonstrated that sufficient HMW DNA could be obtained from a single conifer megagametophyte for this scaffolding. What was surprising was that the majority (nearly 99%) of the DNA extracted from the single megagametophyte was not actually needed since the input requirements of library construction are so extremely modest. Thus this remaining DNA could be used for additional single-molecular physical coverage. Alternatively it could be used as input to the protocol followed in the original sequencing and assembly of *P. lambertiana* (Stevens *et al.* 2016). The implication is that a highly “scaffolded” genome assembly using *only* the DNA from a single MGP is achievable for many conifer species. The only requirement is that the extraction method be optimized for recovering HMW DNA.

Previously, a haploid conifer MGP has been used for robust error correction, cleaning, and compression of both Illumina and PacBio sequences (Zimin *et al.* 2014, 2017; Stevens *et al.* 2016) to form the basis of a high quality genome assembly. The powerful protocol described here augments and extends that approach, and promises much better reference sequences in future applications.
